# Neoadjuvant administration of Semliki Forest virus expressing interleukin-12 combined with attenuated *Salmonella* eradicates breast cancer metastasis and achieves long-term survival in immunocompetent mice

**DOI:** 10.1186/s12885-015-1618-x

**Published:** 2015-09-07

**Authors:** M. Gabriela Kramer, Martín Masner, Erkuden Casales, María Moreno, Cristian Smerdou, José A. Chabalgoity

**Affiliations:** 1 Department of Biotechnology, Instituto de Higiene, Facultad de Medicina, Universidad de la República, (UdelaR), Av. A. Navarro 3051, 11600 Montevideo, Uruguay; 2 Division Gene Therapy, Center for Applied Medical Research, University of Navarra, Av. Pio XII 55, 31008 Pamplona, Spain; 3 IdiSNA, Navarra Institute for Health Research, c/Irunlarrea 3, 31008 Pamplona, Spain

## Abstract

**Background:**

Metastatic breast cancer is a major cause of death among women worldwide; therefore efficient therapeutic strategies are extremely needed. In this work we have developed a gene therapy- and bacteria-based combined neoadjuvant approach and evaluated its antitumor effect in a clinically relevant animal model of metastatic breast cancer.

**Methods:**

2×10^8^ particles of a Semliki Forest virus vector expressing interleukin-12 (SFV-IL-12) and/or 2×10^7^ units of an *aroC*^*−*^ *Samonella* Typhimurium strain (LVR01) were injected into 4T1 tumor nodules orthotopically implanted in mice. Tumors were surgically resected and long-term survival was determined. IL-12 and interferon-γ were quantified by Enzyme-Linked ImmunoSorbent Assay, bacteria was visualized by inmunohistochemistry and the number of lung metastasis was calculated with a clonogenic assay.

**Results:**

SFV-IL-12 and LVR01 timely inoculated and followed by surgical resection of tumors succeeded in complete inhibition of lethal lung metastasis and long-term survival in 90 % of treated mice. The combined therapy was markedly synergistic compared to each treatment alone, since SFV-IL-12 monotherapy showed a potent antiangiogenic effect, being able to inhibit tumor growth and extend survival, but could not prevent establishment of distant metastasis and death of tumor-excised animals. On the other hand, LVR01 alone also showed a significant, although limited, antitumor potential, despite its ability to invade breast cancer cells and induce granulocyte recruitment. The efficacy of the combined therapy depended on the order in which both factors were administered; inasmuch the therapeutic effect was only observed when SFV-IL-12 was administered previous to LVR01, whereas administration of LVR01 before SFV-IL-12 had negligible antitumor activity. Moreover, pre-treatment with LVR01 seemed to suppress SFV-IL-12 antiangiogenic effects associated to lower IL-12 expression in this group. Re-challenged mice were unable to reject a second 4T1 tumor; however 100 % of them could be totally cured by applying the same neoadjuvant combined regimen. To our knowledge, these are the most encouraging results obtained to date in a post-operatory setting using the highly aggressive 4T1 animal model.

**Conclusions:**

SFV-IL-12-based gene therapy combined with *Salmonella* LVR01 neoadjuvant administration has a synergic antitumor effect and may be a promising therapeutic option to prevent and/or eradicate pre-operatory metastasis in locally advanced breast cancer.

**Electronic supplementary material:**

The online version of this article (doi:10.1186/s12885-015-1618-x) contains supplementary material, which is available to authorized users.

## Background

Metastatic breast cancer (MBC), including locally advanced breast cancer (LABC) and distant relapse (DR) are, nowadays, major challenges to oncologists, and to researchers focused on the development of new treatments against these deadly disease situations. Despite advances in early diagnosis and multidisciplinary therapeutic approaches, MBC remains incurable and current main therapies goals range from symptoms palliation to extending survival [1]. In severe cases of LABC, where an underlying invasive disease is often present at the time of diagnosis, neoadjuvant therapies (i.e., administration of therapeutic agents before a main treatment such as mastectomy and/or radiotherapy) are first-line choices [2–4]. Presently, such neoadjuvant therapies relay mostly on cytotoxic chemotherapies which, unfortunately, have limited efficacy, as well as multiple toxic effects. It is, therefore, extremely necessary the development of alternative therapeutic strategies that are more effective against MBC and that can be applicable in neoadjuvancy to treat LABC. In addition, it is also equally important that the new therapeutic approaches are evaluated in clinically relevant animal models of breast cancer.

The murine 4T1 orthotopic model mimics aggressive types of breast cancer since it is rapidly progressive, highly angiogenic and angioinvasive and metastasizes spontaneously from little primary tumors to draining lymph nodes and distant organs, following a metastatic pattern that closely resembles the human counterpart [5–7]. In addition, the carcinogenesis dynamics of 4T1 tumors established in immunocompetent BALB/c mice is comparable to human stage IV type of breast cancer [8]. Moreover, and analogously to human breast cancer, 4T1-derived tumors are poorly immunogenic [6]. In this regard, several mechanistic studies have described the cellular, molecular and soluble tumor-associated and immune-related factors that participate in inhibiting the host immunosurveillance effectors [9–11], making the 4T1 model particularly challenging for evaluating novel strategies aimed at inducing an efficient antitumor immune response. For all these reasons, the investigation and discovery of effective neoadjuvant immunotherapies against the metastatic disease in the murine 4T1 breast cancer model could be of great clinical value.

Gene therapy is a relatively new paradigm in medicine with enormous therapeutic potential. Actually, almost 2000 gene therapy clinical trials (most of them for cancer applications) were registered worldwide, having 96 trials already reached phases II/III to IV, indicating the progress being made with respect to bringing gene therapy closer to the clinical setting [12]. A number of different vectors and delivery systems have been applied; however, viral vectors remain by far the most versatile, popular and effective approach, been used in approximately two-thirds of such clinical trials [13]. We have recently shown that a gene therapy strategy based on the short-term intratumoral (i.t) expression of the potent pro-inflamatory cytokine interleukin-12 (IL-12) expressed from a cytopathic Semliki Forest virus vector (SFV-IL-12) inhibits tumor growth and extends survival in a transgenic mice model of hepatocellular carcinoma (HCC) [14]. Moreover, this vector was also efficient in reducing tumor volume and inducing T cell-mediated responses against HCC spontaneously developed in woodchucks chronically infected with an hepadnavirus very similar to hepatitis B virus, a situation that closely resembles HCC disease in humans [15]. In addition, SFV-IL-12 was proven to have a stronger antitumor potential compared to other viral and non-viral-based IL-12 expression systems *in vivo* due to the strong SFV-mediated induction of apoptosis and activation of type-I interferon responses specifically in the tumor [14, 16, 17]. When searching whether the therapeutic spectrum of this vector was expanded to breast cancer, we found only one previous report where a similar SFV-IL-12 construct was tested in the 4T1 model. There, a significant reduction in primary tumor size as well as in the percentage and number of lung metastases was observed after several i.t. administrations of high amounts of vector [18]. However, no information was provided about long-term survival (i.e, at least 6 months after treatment) and/or anti-metastatic effect of the therapy in tumor-excised mice, which would be a more clinically relevant condition, due to the fact that, in general, patients have their primary tumor surgically removed.

On the other hand, bacteria-based therapies are a modality of increasing interest in anticancer immunotherapeutic research due to the capability of many auxotrophic mutants to restrict their growth to the rich nutrient milieu tumor interior, inducing cell destruction and liberation of tumor antigens, as well as *in situ* inflammation, triggering a versatile immune response upon bacterial infection [19–21]. It has also been shown that facultative anaerobic bacteria, like attenuated *Salmonella enterica* serovar Typhimurium (*S*. Typhimurium) and genetically modified *Escherichia coli* strains, elicit antitumor potential against breast cancer models, especially if combined with plasmids that express cytotoxic inducing factors or immunostimulatory cytokines [22–26]. However, similarly as for IL-12-based gene therapies, these studies were mostly focused on evaluating the antitumor effect of bacteria against primary tumors; and so far there are no data reporting complete remission of 4T1 primary tumors or total inhibition of lung metastasis in operated mice.

Therefore, and in order to go a step forward towards a possible clinical application of a gene- and bacteria-based neoadjuvant therapy for MBC, we have evaluated the anti-metastatic potential of the i.t. administration of SFV-IL-12 and/or *aroC*^*−*^ attenuated *S.* Typhimurium strain LVR01 [27] into 4T1 primary tumors followed by their surgical resection. Our data showed a clear synergic antitumor effect of the combined therapy compared to SFV-IL-12 and LVR01 monotherapies, achieving metastasis-free and long-term survival in 90 % of treated animals. Moreover, we observed that the efficacy of the combined therapy radically depends on the order in which both factors were administered; suggesting the existence of specific mechanisms underlying the observed synergic therapeutic effect.

## Methods

### Cell lines and bacteria

Mouse breast carcinoma 4T1 cell line was obtained from American Type Culture Collection (ATCC-CRL-2539™) and grown in high Glucose (4.5 g/l) Dulbecco’s Modified Eagle’s Medium (DMEM) with stable Glutamine, Sodium Pyruvate and 3.7 g/l NaHCO_3_ (PAA Cell Culture Company P04-04510) supplemented with 10 % Fetal Bovine Serum (FBS, GE Healthcare). Chinese hamster ovary cells CHO-K1 (ATCC- CCL-61™) used to produce the viral vectors were cultured in F12K Medium (ATCC- 302004) supplemented with 10 % FBS. The BHK-21 cell line (ATCC- CCL-10™) used to titer SFV-based vectors, was cultured in Glasgow Minimum Essential Medium (Invitrogen, Carlsbad, CA) supplemented with 5 % FBS. All cell lines were grown at 37 °C in a humidified CO_2_ incubator and passaged when confluent using a 0.25 % Trypsin 0.53 mM EDTA solution. The live attenuated *Salmonella* strain employed in this study, LVR01, was constructed earlier by introducing a null deletion into the *aroC* gene of the parental canine *S*. Typhimurium isolate, P228067 [27]. Bacteria cultures were grown at 37 °C in Luria-Bertani (LB) media shaking at 200-250 rpm to OD_600_ = 1.5 and stored at -80 °C in 17 % glycerol stocks until used.

### Viral vectors production

Recombinant SFV RNAs carrying *IL-12* or *lacZ* genes were amplified *in vitro* from pSFV-enhIL-12 [28] or pSFV-LacZ [29] template plasmids, respectively, using the SP6 DNA dependent RNA polymerase reaction and co-electroporated into CHO-K1 cells with SFV-helper-S2 and SFV-helper-C-S219A RNAs (which provided *in trans* the envelope and capsid SFV proteins, respectively), as described previously [30]. Electroporated cells were incubated at 33 °C for 48 hs, supernatants were collected and SFV viral particles (vp) were purified by ultracentrifugation through a 20 % sucrose cushion at 100,000 g during 90 min. Titers of viral vector stocks were determined in infected BHK-21 cells by indirect immunofluorescence using a rabbit polyclonal antiserum specific for the nsp2 subunit of SFV replicase as described [31]. Titers of 1.0x10^11^ for SFV-IL-12 and 1.2×10^11^ vp/ml for SFV-LacZ were obtained.

### Breast cancer model and treatments

All experimental procedures involving animals were approved and performed in accordance with our University’s Ethics Commission for Animal Experimentation (CHEA, UdelaR). Six to 8 weeks old female BALB/c mice (DILAVE, Uruguay) were inoculated in the 4th right mammary fat pad with 70,000 4T1 cells resuspended in 50 μl phosphate saline buffer (PBS). Ten days after cell implantation, 100 % mice showed palpable tumors and were homogenously divided into the different treatment groups. Fifty μl (in PBS) of the corresponding treatments were injected into tumors using a sterile 1 ml syringe with 27G needle. Tumor size was periodically measured with a precision caliper and its volume was calculated by the ellipsoid formulae (major diameter × minor diameter^2^ × 0.5). Surgical removal of tumors was performed essentially as described [5, 8]. In addition, the proximal draining lymph node was also carefully removed along with the adjoining abdominal fat. Wounds were closed using a sterile 19 mm 3/8 nylon monofilament suture (HAD, China). Paracetamol (1 mg/ml) dissolved in drinking water was given to animals for 24 hs to aid the post-operatory recovery. Survival was monitored regularly, and animals were euthanized if found moribund during the observation period. Dead mice were autopsied to check for the presence of lung metastasis.

### Immunohistochemistry

Cryosections (5 μm thick) of tumors pre-infected with 2×10^7^ cfu (colony forming units) were fixed with 4 % paraformaldehyde (PFA), permeabilized with 0.2 % Tween 20 in PBS and blocked with 1 % BSA in PBS prior to staining using a specific rabbit anti-*Salmonella* lipopolysaccharide (LPS) O Poly A antiserum (BD Biosciences). Detection was performed with secondary goat anti-rabbit Alexa Fluor® 555-conjugated antibody (Invitrogen). Cell membranes were stained using wheat germ agglutinin (WGA) conjugated to Oregon Green® 488 or Alexa Fluor® 350 (Life Technologies). The third channel of fluorescence was occupied by 4’,6-diamidino-2-phenylindole (DAPI, Invitrogen) or anti-Gr1 antibody conjugated to Alexa Fluor® 488 (BD Biosciences) to detect cell nuclei or granulocytes, respectively. Acquisition and processing of images were carried out using a Nikon® Ti-S epifluorescence inverted microscope and analyzed with the NIS-Elements software (Nikon).

### Lungs metastasis

The number of 4T1 cells that reach the lungs during its spontaneous metastatic process can be quantified by a standard clonogenic assay due to their inherent resistance to 6-thioguanine treatment [6, 7]. To this end, mice were sacrified 35 days after tumor cells implantation, lungs were isolated and finely minced with sterile scissors and digested in a 5 ml PBS solution containing 20 mg Collagenase type I (230 u/mg, GIBCO™ 17100-017) and 50 μg DNAseI (Sigma-Aldrich) for 1 h at 37 °C on a rotating wheel. After incubation, 5 mM EDTA was added to stop the enzymatic reaction and the tissue was homogenized by pippeting several times. The homogenate was washed in 10 ml PBS and samples were filtered through a 70 μm Falcon cell strainer (BD Biosciences) to obtain a clear solution. This solution was washed twice in DMEM by 1500 rpm centrifugation at 4 °C. The cell pellet was resuspended and serially diluted in 6-well tissue culture plates containing DMEM supplemented with 10 % FBS and 1× Antibiotic/Antimycotic Solution (Capricorn Scientific AAS-B) plus 60 μM 6-thioguanine (Sigma Aldrich). Tumor cells formed foci within 10-14 days. Cell clones were fixed with PFA 4 % and stained with 0.03 % methylene blue solution for macroscopic counting. Resistant clonogenic metastasis were calculated on a per-organ basis as described [8].

### Cytokine serum levels

Blood samples were obtained from the mice facial vein and serum was recovered by centrifugation at 10,000 rpm for 10 min and stored at -80 °C until protein measurement. Murine IL-12 and IFN-γ levels were quantified by ELISA (Enzyme-Linked ImmunoSorbent Assay) using Op-tEIA Mouse IL-12 and IFN-γ Sets (BD Biosciences), respectively, and according to the manufacturer’s instructions.

### Statistical analysis

GraphPad Prism version 6.0 software was employed to perform the statistical analyses. One way ANOVA was used to assess the difference between groups in multiple comparisons regarding one variable and Student’s *t*-test was applied to evaluate the differences between selected pair of groups. Survival curves obtained by the Kaplan-Meyer method were statistically analyzed using the Log-rank test. Data were considered significant when *p* values were ≤0.05.

## Results

### Early lung metastases accompany 4T1 tumor growth and could mimic LABC upon surgical resection of primary tumors

**Fig. 1:**
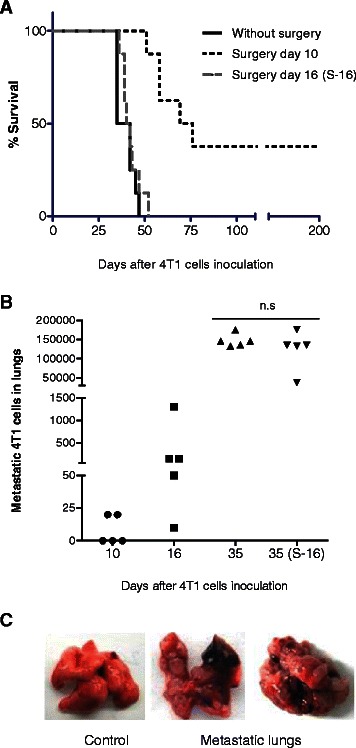
Effect of 4T1 breast cancer metastases in animals that underwent tumor surgery. Tumor cells were orthotopically implanted in mice mammary fat pads (day 0). Primary tumors were left without surgery or removed by surgical excision, 10 or sixteen (S-16) days later. **a** Kaplan–Meier plot shows the survival rate of the indicated groups (*n* = 8). **b** Additional tumor-bearing or tumor excised mice were sacrificed at 10, 16 or 35 days after 4T1 cells inoculation, lungs were extracted and processed and the number of lung metastases were quantified as described in Methods. 35 (S-16) refers to a group of mice that underwent surgery at day 16 and were checked for lung metastases 19 days later (i.e. 35 days after 4T1 tumor implantation). **c** Lungs extracted from a healthy control mouse (Control) and from moribund mice showing numerous metastatic nodules (Metastatic lungs). Non-significant (n.s.)

Breast cancer aggressiveness and poor prognosis is commonly associated with metastasis occurrence to distant organs, such as lungs, liver and bones. In this regard, it is well known that early resection of primary tumors contributes to control metastasis; however clinical outcome depends on many factors, including growth dynamics and metastatic characteristics of these tumors. In order to correlate the 4T1 breast cancer model with clinical cases of LABC in respect to its metastatic progression, we evaluated the therapeutic impact of primary tumor resection at different time points. Small tumors (4-5 mm diameter) could be homogeneously detected 10 days after orthotopic implantation of 7×10^4^ 4T1 cells and by day 16 tumors reached 6–7 mm diameter, still a feasible surgically size. Thus, we defined days 10 and 16 post-4T1 implantation as early and middle-early time points for tumor resection, respectively. Control tumor-bearing mice have a life span of 44 ± 5 days after 4T1 cells implantation (Figs. 1 and 8b). Animals whose tumors were surgically resected at day 10 showed an improved overall survival (with 40 % mice surviving more than 6 months), while 100 % animals operated at day 16 died at the same rate as non-operated mice (Fig. 1a). In agreement with these data, lung micro-metastatic cells were present at day 10 in 40 % of the animals, while the rest were metastasis-free; however, 6 days later (day 16), 100 % animals presented a variable number (10-1300) of lung micro-metastasis (Fig. 1b). Middle-early excised tumor mice and non-operated controls showed comparable life span and a similar amount of established lung metastasis (in the order of 10^5^ 4T1 tumor cells/lungs) 35 days after tumor implantation (Fig. 1b) suggesting that both facts are associated. The autopsy of moribund mice revealed in all cases the presence of numerous macroscopic metastatic foci and a massive distortion of both lungs (Fig. 1c), accompanied by severe breathing difficulties at the time of death. These data confirmed the determinant role of the metastatic disease in the survival outcome of the 4T1 model of breast cancer [6]. In addition, non-survivor animals occasionally presented visible metastatic tumor nodules in liver, lymph nodes, spleen and/or heart (not shown). It is important to note, that just a small number of 4T1 metastatic cells (around 10–20, Fig. 1b) are sufficient to progress to a severe lung metastatic disease in the absence of primary tumors, indicating that these tumor cells may be particularly resistant or indifferent to the host immune system in order to persist. Due to the early metastatic dissemination and their growing characteristics, we conclude that the 4T1-tumor model could reflect most aggressive clinical cases of LABC. Moreover, middle-early tumor-excised mice would represent an excellent model of minimal residual disease (MRD) for therapeutic intervention against metastatic disease, since 100 % of animals develop lung metastasis and do not survive in the long-term. Our data also point out to a temporal window between days 10 and 16 (initiation - establishment of the metastatic process) for evaluation of novel neoadjuvant strategies (prior to surgical removal of primary tumors) aimed at preventing and treating lethal MRD.

### Intratumoral administrations of SFV-IL-12 inhibit tumor growth, reduce angiogenesis and prolong survival in 4T1 tumor-bearing mice

**Fig. 2:**
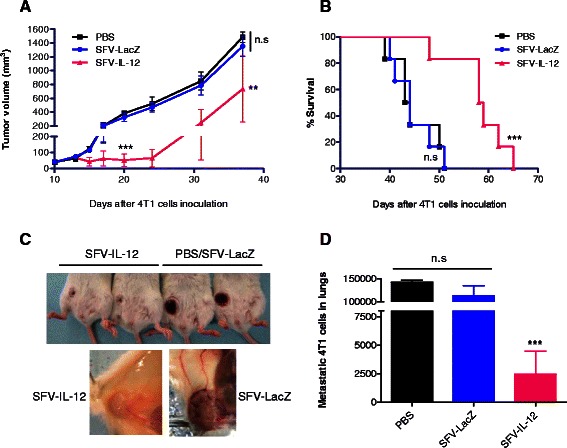
Antitumor effect of SFV-IL-12 in 4T1 tumor bearing mice. Two doses of SFV-LacZ or SFV-IL-12 (2×10^8^ vp in 50 μl PBS) were i.t. injected at days 10 and 13 after 4T1 cells inoculation. An equivalent volume of PBS was administered to control animals **a** Tumor size progression was measured every 3-6 days and mean tumor volumes were calculated for each group (*n* = 6). **b** Kaplan–Meier plot shows the survival rate of the same mice. **c** Pictures of representative tumors 21 days after 4T1 cells inoculation showing the external aspect (upper panel) and interior tumor region (lower panel). A reduced volume and vasculature was observed in treated mice compared to control mice. **d** An additional group of treated and control animals were sacrificed 35 days after tumor cells inoculation and 4T1 lung metastases were quantified as described in Methods. Non-significant (n.s), *p*< 0.01 (**); *p*< 0.001 (***)

We and other groups have shown that the amount of IL-12 expressed at the tumor site using different viral vectors is critical for tumor regression in a number of cancer models [14–16, 18, 28, 32]. In order to evaluate the therapeutic potential of SFV-IL-12 against breast cancer, we took into consideration all these previous experiences, and injected a relatively high dose of SFV-IL-12 (2×10^8^ vp) at two early stages of the disease: the first dose was given at day 10 and the second dose was given at day 13 after 4T1 implantation (Fig. 2). Tumor growth was periodically measured and animals were monitored for survival outcome. Similarly, control groups received two i.t. doses of an SFV vector expressing β-galactosidase (SFV-LacZ) or PBS. As shown in Fig. 2a, treatment with SFV-IL-12 caused a significant inhibition of tumor growth prolonged until days 20-25. At this stage, control animals showed large ulcerated and irrigated tumors, whereas SFV-IL-12 treated tumors were small and whitish, due to a visible reduction of vasculature (Fig. 2c). These data are consistent with the described antiangiogenic effect of IL-12 in 4T1 tumors and in other IL-12-treated tumor models [18, 32–35] indicating the *in vivo* activity of this cytokine. The survival of SFV-IL-12 injected animals was significantly extended compared to the SFV-LacZ and PBS control groups, although no animals showed long-term survival (Fig. 2c). Moreover, a significant lower number of lung metastasis was observed at day 35 in SFV-IL-12 treated mice (Fig. 2d), suggesting that their extended survival rate may be a consequence of the reduced metastatic burden at a time point when untreated animals start to die. Together, these results show that SFV-IL-12 induce a marked local antiangiogenic effect in implanted 4T1 tumors, which may contribute to inhibit primary tumor growth for a limited period of time, as well as to reduce dissemination of metastasis in the organism, prolonging consequently animal survival. However, SFV-IL-12 monotherapy, at least at the employed dose regimen, was insufficient to achieve complete tumor remission and to prevent the spread of 4T1 lung metastasis.

### Surgical resection of tumors after SFV-IL-12 administrations achieves long-term survival in 20 % mice

**Fig. 3:**
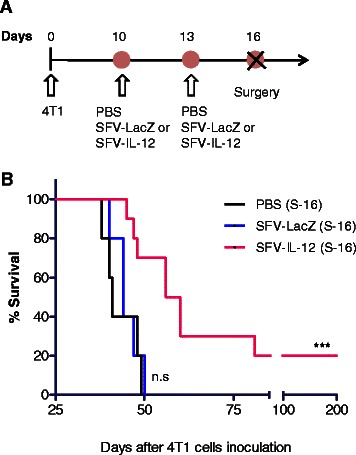
Neoadjuvant effect of SFV-IL-12. **a** Schematic representation of the therapeutic protocol performed in this study. Two doses of SFV-LacZ, SFV-IL-12 or PBS were i.t. injected as in Fig. 2. Three days later (i.e. day 16 after 4T1 cells inoculation), treated tumors were surgically removed (S-16). **b** Kaplan–Meier plot shows the survival rate of treated and control mice (*n* = 5-10). Non-significant (n.s), *p*< 0.001 (***)

In order to mimic frequent management of patients with LABC, we have implemented a therapeutic protocol that included surgical removal of primary tumors after a preliminary treatment. Unlike conventional neadjuvant systemic approaches, here we performed local administration of the therapeutic agent (Fig. 3a). Two doses of SFV-IL-12 were injected inside the tumor mass at days 10 and 13 after 4T1 cells inoculation. Tumors were surgically removed 3 days later (day 16) and survival outcome was compared to SFV-LacZ or PBS treated mice. As shown in Fig. 3b, the overall survival of SFV-IL-12 treated animals was significantly higher than that of controls. In addition, 20 % of long-term survivors were observed in the IL-12-based neoadjuvant post-operatory setting. All of the non-survivor animals experienced breathing difficulties before death and showed numerous macroscopic lung metastases as was previously observed in untreated mice (see Fig. 1c). In contrast, mice that survived at the end of this study did not present any visible metastasis. However, the fact that long-term survival was only achieved in a small percentage of treated mice indicated that our strategy to treat this type of cancer needed to be improved. Based on several studies that report the benefit obtained when combining IL-12-based gene therapy with complementary strategies or drugs [36–40], we next decided to combine SFV-IL-12 with another biological agent that could collaborate in inducing a more efficient response against the metastatic disease.

### Synergistic anti-metastatic effect of combined SFV-IL-12 and *Salmonella* LVR01 neoadjuvant therapy achieves long-term survival in 90 % treated mice

**Fig. 4:**
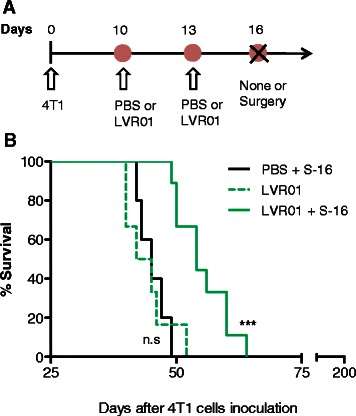
Antitumor effect of LVR01 in 4T1 tumor bearing and tumor-excised animals. **a** Schematic representation of the therapeutic protocol performed in this study. Two doses of LVR01 (2×10^7^ cfu in 50 μl PBS) or 50 μl PBS were i.t. injected at days 10 and 13 after 4T1 cells inoculation. In the indicated groups, the treated tumors were surgically removed at day 16 (S-16). **b** Kaplan–Meier plot shows the survival rate of treated and control mice (*n* = 5-9). Non-significant (n.s); *p*< 0.001 (***)

**Fig. 5:**
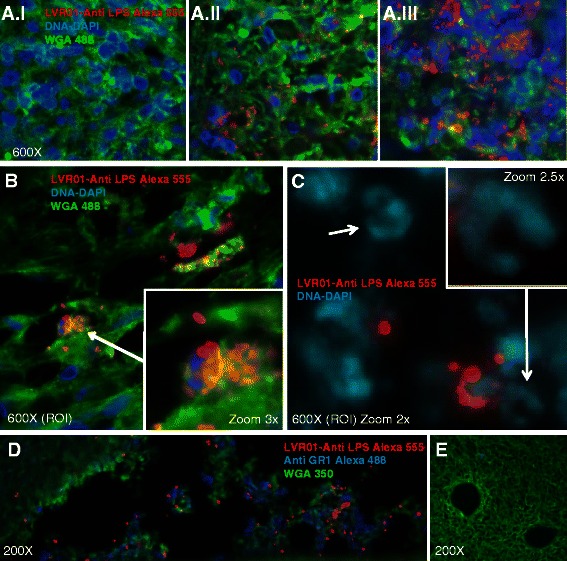
Microscopic characteristics of LVR01 infected tumors. 2×10^7^ cfu were inoculated into 4T1 tumors. Three days later, tumors were extracted, crio-preserved and analyzed. In figures **a**-**c** LVR01 is stained in red (AntiLPS Alexa 555), DNA in blue (DAPI) and cell membranes in green (WGA 488). In **d**-**e**, Gr1+ cells are shown in blue (Gr1-Alexa 488) and cell membranes are shown in green (WGA350). **a** Tumor regions without bacteria (I), or invaded with low (II) or high (III) density of LVR01 show a dose-dependent tissue distortion effect. **b** Bacterial conglomerates (*arrow*) indicate possible intracellular replication. **c** DNA staining of infected zones reveals nuclear morphologies characteristic of myeloid cells (*arrows*) in the proximity of LVR01 accumulation. **d** Gr1^+^ cells are present in the proximity of bacterial infected zones **d** Absence of granulocytes in tumoral regions without S*almonella*

We hypothesized that live attenuated *Salmonella* could be a good complement for IL-12-based antitumor therapy due to the intrinsic immune-stimulatory properties and the better antitumor responses observed when administering bacteria carrying plasmids that express pro-inflammatory cytokines (reviewed in [41]). Additionally, a recent related study of our group has shown that i.t. administrations of *Salmonella* LVR01 generate a considerable dose-dependent antitumor effect in a mouse model of B-cell lymphoma [42]. Despite this result, when two doses of LVR01 were inoculated into primary 4T1 tumors, no survival improvement was seen in tumor-bearing animals (Fig. 4). Surgical removal of the tumor after LVR01 inoculations led to a significant death delay, although no animals survived for the long-term (Fig. 4). In this case, all mice died with large macroscopic lung and disseminated metastasis, indicating that LVR01 alone was insufficient to control the 4T1 metastatic process. Nevertheless, the inoculated bacteria were able to invade tumor cells and induce local microscopic tissue distortion and granulocyte infiltration (Fig. 5), persisting inside the tumor mass for several days (see Additional file 1).

**Fig. 6:**
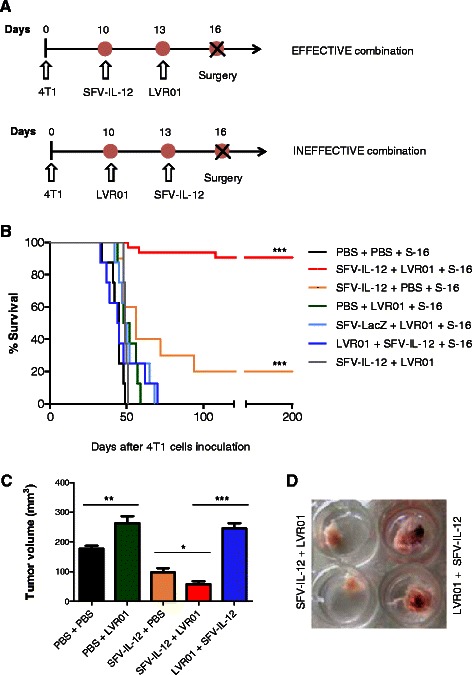
Combined neoadjuvant antitumor effect of SFV-IL-12 and LVR01. One dose of SFV-IL-12 or SFV-LacZ (2×10^8^ vp in 50 μl PBS), or LVR01 (2×10^7^ cfu in 50 μl PBS), or 50 μl PBS was i.t. injected at days 10 and/or 13 after 4 T1 cells inoculation. In most groups, treated tumors were surgically removed at day 16 (S-16). **a** Schematic representation of the therapeutic protocol for the combinations relevant to this study. **b** Kaplan–Meier plot shows the survival rate of the indicated groups of mice. The total number of animals included in the SFV-IL-12 + LVR01 + S-16 group was 31 (consecutive experiments) and the 28 survivor mice were employed in the re-challenge study presented in Fig. 8. For the rest of the groups *n* = 8-10. **c** Tumor size was measured before surgery (day 16) and mean + SD tumor volumes were calculated for each group. **d** Representative images of treated tumors excised 16 days after 4 T1 cells inoculation. *p*< 0.05 (*); *p*< 0.01 (**); *p*< 0.001 (***)

**Fig. 7:**
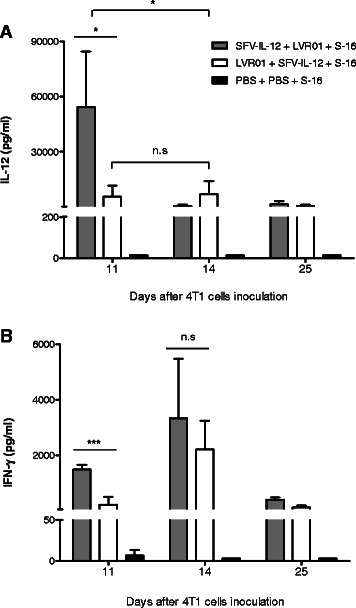
Expression of cytokines in treated mice. Blood samples were collected the next day after administration of the combined treatments presented in Fig. 6 (days 11 and 14) and 9 days after surgery (day 25) and serum levels of **a** IL-12 and **b** IFN-γ were measured by ELISA. Non-significant (n.s); *p*< 0.05 (*); *p*< 0.001 (***)

On the other hand, when SFV-IL-12 (day 10) was combined with LVR01 administration (day 13) followed by surgical removal of the treated tumor (day 16, Fig. 6a), a clear synergy in the antitumor action was observed, evidenced by 90 % long-term survival without metastasis. However, if treated tumors were not resected, all mice died, indicating that this combined therapy only works in neoadjuvancy (Fig. 6b). Interestingly, we found that the order of the injected factors dramatically affected the antitumor response, since the administration of LVR01 prior to SFV-IL-12 did not show any synergy and all animals died at similar rate than control groups (Fig. 6a and b). It is worth to note, that at the time of surgery, tumors receiving only LVR01 or the ineffective LVR01 + SFV-IL-12 combination showed a bigger size compared to SFV-IL-12 alone or SFV-IL-12 + LVR01 treated animals (Fig. 6c). In addition, inhibition of vasculature was observed with effective SFV-IL-12 + LVR01, but not with the LVR01 + SFV-IL-12 combination (Fig. 6d). Moreover, we found that IL-12 expression was significantly reduced in mice receiving LVR01 prior to SFV-IL-12 (day 14, Fig. 7a), while a higher amount of IFN-γ (a main mediator of IL-12 activity) was induced earlier in the effective combination (day 11, Fig. 7b), indicating that the right levels and dynamics of both cytokines expression may be associated with the curative effect of the combined therapy. In this regard, we also observed a significant increase of CD8^+^ and CD4^+^ T cells in draining lymph nodes isolated from the effective LVR01 + SFV-IL-12 combined group (see Additional file 2). Overall, these data demonstrate that the synergic neoadjuvant action of SFV-IL-12 and *Salmonella* LVR01 prevent and eradicate breast cancer metastasis that could be disseminated in the organism before surgical removal of the tumors. Our observations point out to the importance of reducing angiogenesis and inducing an efficient immune response at early stages of the disease.

### Re-challenged mice efficiently respond to a second SFV-IL-12 + LVR01 neoadjuvant treatment

**Fig. 8:**
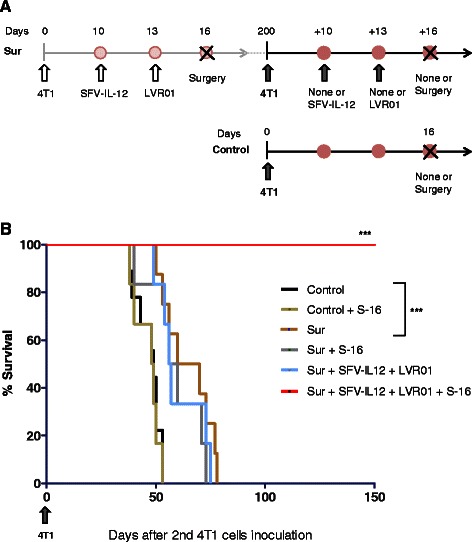
Re-challenge study and re-treatment assay. **a** Schematic representation of the performed protocols. The mice that had survived for 200 days in the experiment described in Fig. 6 were challenged with 10.000 4T1 cells implanted in the forth left mammary fat pad (opposite to the site where the first tumor was implanted). Ten days later tumors were already palpable and some of these animals were left without further treatment (Sur) or underwent tumor surgery 16 days later (Sur + S-16), or received SFV-IL-12 (2×10^8^ vp in 50 μl PBS) at day 10 and LVR01 (2×10^7^ cfu in 50 μl PBS) at day 13 (Sur + SFV-IL-12 + LVR01), or received this last treatment followed by surgery (Sur + SFV-IL-12 + LVR01 + S-16). A healthy and untreated group of animals with similar age were equally inoculated with 10.000 4T1 cells and left without further treatment (Control) or underwent surgery (Control + S-16). **b** Kaplan–Meier plot shows the survival rate of the indicated groups of mice (*n* = 6-9). Survival days were counted since the second (re-challenged) 4T1 inoculation. *p*< 0.01 (**); *p*< 0.001 (***)

As shown above, surgery of primary tumors performed 16 days after 4T1 cells inoculation did not prevent lung metastasis formation and animal death, whereas an efficient long-term antitumor effect was observed after receiving the right neoadjuvant combined therapy. This data suggests that the mice cured from metastatic disease may have developed a systemic antitumor immunity, which, in theory, could protect them from a second 4T1 cells challenge. To test this hypothesis, we inoculated 10^4^ 4T1 cells in the left abdominal mammary gland of cured animals 6 months after the first 4T1 tumor implantation (Fig. 8a). Contrary to our expectations, all of these animals developed mammary tumors and died, although with a significant slower rate than control naïve mice that received an equal dose of 4T1 cells (Fig. 8b), indicating that specific antitumor immunity might be elicited. However, removal of the primary tumor at day 16 post-inoculation (performed in order to reduce tumor cells burden in the organism) did not improve survival, suggesting that this elicited immune response was too weak to protect these mice from a secondary challenge. On the other hand, treatment with SFV-IL-12 and *Salmonella* LVR01, when followed by excision of the tumor, again showed a very potent antitumoral effect, achieving 100 % of long-term survival (Fig. 8b). These data indicate that although the combined therapy does not induce an efficient long-lasing protection against 4T1 tumors, it allows the use of the same treatment to cure a secondary mammary tumor in the same animal, a situation that could be clinically relevant to treat patients who suffer from relapse.

## Discussion

Neoadjuvant therapies aim to reduce the size or extent of primary tumors before radical intervention, as well as to eliminate early disseminated micro-metastasis in locally advanced malignancies, such as LABC [2]. In the present study, we have evaluated the efficacy of a combined neoadjuvant gene therapy approach against the highly metastatic 4T1 mouse model, which we have correlated here with severe LABC type of cancers (Fig. 1). Our neoadjuvant approach is based on the use of IL-12, a potent immunostimulatory cytokine with strong antiangiogenic activity [32, 35, 43] in combination with LVR01, an attenuated auxotrophic mutant of *S*. Typhimurium [27, 42] as co-adjuvant factor. To allow IL-12 expression into the tumor mass we employed a SFV-based vector encoding IL-12 genes (SFV-IL-12), which was previously shown to express IL-12 (locally and systemically) upon i.t. administration [14, 28]. In this construct, each IL-12 subunit (p35 and p40) is driven by an independent SFV subgenomic promoter fused to the SFV capsid translation enhancer achieving higher expression levels and stronger antitumor activity compared to adenoviral vectors engineered to express IL-12 [16, 28]. SFV-based vectors have also the capability of inducing apoptosis of infected cancer cells, allowing the release of tumor-associated antigens that could be taken up and cross-presented by antigen-presenting cells [14, 36, 44, 45]. In addition, SFV-IL-12 achieves transgene expression after multiple i.t. administrations and triggers efficient antitumor immune responses in different cancer models [14–16, 18, 28, 46]. *Salmonella* LVR01 was also demonstrated to induce death of infected tumor cells and to trigger innate and adaptive immune responses after multiple i.t. administrations [42]. These responses are associated to Toll-like receptors recognition of pathogen associated molecular patterns (PAMPs) in the bacteria, such as lipopolysaccharide (LPS), teichoic acid, peptidoglycan, and bacterial CpG DNA [41, 47]. Although the LVR01 bacterial strain has been extensively tested as carrier for heterologous antigens for human and veterinary vaccination applications [48–50], its use as anticancer agent is relatively recent [42, 51] and therefore is still an open area for investigation. Still, the use of *Salmonella* for anticancer therapies, either as monotherapy or combined, has received extensive consideration in the last decade, showing different degree of effectivity in a number of animal models of major human cancer types including breast cancer [52–58]. Particularly, a *Salmonella* A1-R-based monotherapy was recently reported with promising results in a 4T1 brain metastatic model [59], although these experiments were conducted in nude mice and long-term protection was not evaluated. In any case, to date only a few approaches using attenuated strains of *Salmonella* have moved into initial clinical trials and none of them have progressed into large phase II clinical trials, paving the way for the search of alternative combined therapies.

Our data showed a clear reduction of tumor blood supply, delay of tumor growth, lower number of lung metastasis and a better survival in SFV-IL-12 treated mice compared to PBS and SFV-LacZ controls. Even though, only 20 % of animals survived long-term after receiving one or two i.t. doses of SFV-IL-12 in the neoadjuvant setting, indicating that an increased dose regimen of SFV-IL-12 monotherapy did not seem to augment its antitumor effectiveness (Figs. 3 and 6, respectively). In this regard, a previous report showed inhibition of primary tumor growth after 6 consecutive i.t. injections of a similar SFV-IL-12 vector in 4T1 tumor-bearing mice, inferring an effective antitumor response using SFV-IL-12 alone [18]. However, these studies were followed up only until day 22 (when mice were sacrificed in order to measure the presence of lung metastasis), a time period where we also observed tumor growth inhibition. Nevertheless, in our long-term studies, all tumors recovered their growth dynamics after day 25-30 and animals died between days 50 and 65 due to metastatic disease, indicating that IL-12 alone was efficient in delaying tumor growth and death, but insufficient to cure these animals. In accordance with our data, the authors also observed metastasis reduction and a potent antiangiogenic effect of SFV-IL-12 in 4T1 injected tumors, a fact that may delay nutrient access to the tumor mass and metastasis spread [18]. The antiangiogenic contribution of IL-12 together with its immunomediated antitumor properties were consistently studied and reported previously [32, 34, 35, 43, 60], as well as its limitations as a single antitumor agent [36]. For that reason, in this work we focused on enhancing the SFV-IL-12–based therapy by combining it with an appropriate “therapeutic companion” in order to achieve long-term cure, which was the main goal of the present study.

A number of different immunomodulatory factors and tumor-specific antigens have shown synergistic effects with IL-12-based therapies in different cancer models [36, 60–62]. However, most of these studies were aimed at evaluating antitumor efficacy against primary tumors, without assessing their effects in neoadjuvancy. In this regard, a relevant study performed in a lung alveolar metastatic model, showed that i.t. administration of IL-12-encapsulated biodegradable microspheres combined with granulocyte-macrophage colony-stimulating factor (GM-CSF), followed by surgical removal of tumors, achieved significant post-operatory long-term survival and reduction of metastasis in animals receiving the combined treatment, compared to each monotherapy [37]. These data indicate that *in situ* IL-12-based neoadjuvant treatments could help to prevent post-operatory dissemination of the metastatic disease, and, together with other studies, confirm that antitumor IL-12-based therapies could benefit from combinations with other cytokines, antibodies or chemotherapy [36–40]. We hypothesized that live attenuated *Salmonella* could be a proper IL-12 co-adjuvant due to the bacterial intrinsic immune-stimulatory and antitumor properties, its easy and cheap high-scale production, and its enhanced therapeutic activity observed when transformed with plasmids expressing immune-stimulatory cytokines ([41] and references therein). Recently, Grille et al, showed an improved survival outcome in mice receiving multiple i.t. doses of *Salmonella* LVR01 in the A20 lymphoma model [42]. However, in the 4T1 breast cancer model, we did not see any therapeutic response in tumor-bearing animals treated solely with LVR01, confirming the previously inferred resistance of 4T1 tumors to immunotherapies [13], although a significant increase in the overall survival was achieved in neoadjuvancy. Anyhow, we detected the infiltration of granulocytes to the tumor site, a relevant observation, since it was suggested that this cell population (specially neutrophyls) is involved in *Salmonella*-mediated therapeutic effect [42, 63]. In addition, LVR01-treated tumors showed microscopic distortions, probably due to the bacterial replication and destruction of infected cells. It is important to remark that all 4T1 tumors treated with LVR01 alone (either with 1 or 2 doses) experienced a significant increase in size, which is consistent with a strong inflammatory reaction and observed vascularization. Consequently, these tumors were more difficult to resect than their SFV-IL-12 counterparts, because they got strongly attached to the peritoneum and skin borders. This fact made the evaluation of a multiple dose regimen protocol surgically impracticable.

We discovered an impressive synergistic effect when a single i.t. dose of SFV-IL-12 (2×10^8^ vp given at day 10 after 4T1 cells implantation) was followed by a single i.t. dose of LVR01 (2×10^7^ cfu given at day 13) prior to surgical removal of the treated tumor (day 16). Here, 90-100 % treated animals survived for a time period that could be considered life-long in mice (total 350 days; Figs. 6 and 8). However, to our surprise, if LVR01 was administered before SFV-IL-12, the combined antitumor synergy was completely lost in our neoadjuvant experimental setting. The reason by which this synergy operates or can be abolished may involve multiple mechanisms, since the type of vector, cytokine and bacteria employed in this study are able to trigger a diverse spectrum of effects and pathways [17, 18, 28, 42, 45] and therefore, their relative contribution will require further investigations. So far, and based on our data, we could mainly speculate that i.t administration of SFV-IL-12 allow sufficient IL-12 expression to activate antiangiogenic responses contributing to inhibit primary tumor growth, and in this favorable context (which might converge with an adequate amount and type of SFV-IL-12-induced immune mediators [17, 34]), the addition of a potent adjuvant, like LVR01, can probably promote a systemic response able to eliminate disseminated micro-metastases in the organism, something that is not achieved by SFV-IL-12 or LVR01 alone. In accordance with the higher expression levels of IL-12 observed in the SFV-IL12 + LVR01 *vs* LVR01 + SFV-IL12 treated animals, also a significantly higher production of IFN-γ was achieved in the former group at early time points, promoting the idea that the better therapeutic outcome in this group could be associated to a T helper 1 primed response timely induced for which an early source of IFN-γ is strictly required [64]. Indeed, previous studies demonstrated that CD8^+^ cytotoxic T cells are major players in antitumor responses triggered by SFV-IL-12 either alone or combined with a proper agonist [28, 36, 46]. Consistently, we obtained initial data showing a higher amount of CD8^+^ T cells in draining lymph nodes isolated from SFV-IL-12 + LVR01 compared to LVR01 + SFV-IL-12 treated animals. The fact that synergy is not observed when LVR01 is given before SFV-IL-12 suggests that the first agent is not as efficient as SFV-IL-12 to prime an adequate microenvironment and efficient immune responses, or alternatively, that LVR01 infection could affect posterior SFV-IL-12 tumor cells transduction. In any case, we found that tumor pre-infection with LVR01 resulted in a significant decrease of SFV-IL-12-mediated expression of IL-12, which would explain the lack of IL-12-mediated antiangiogenic effect in treated tumors, as well as the reduced IFN-γ levels and CD8^+^ T cells in these animals. Overall, our data suggest the importance of reducing angiogenesis and inducing an efficient immune response at early stages of the disease in order to achieve a potent therapeutic effect.

The lack of protection against a secondary challenge may indicate that our therapeutic procedure does not stimulate an efficient long-lasting antitumor immunity. Nevertheless, we found that the application of same treatment (combined adjuvant therapy followed by surgical removal of primary tumors) is equally effective to cure animals when used into re-challenged mice, a finding that we believe could be clinically relevant to treat patients who suffer from relapse. Thus, this combined therapy may be worth of considering for clinical trials.

In summary, we have described a promising neoadjuvant therapeutic strategy to treat a highly aggressive type of breast cancer. To our knowledge, this is the first experimental protocol reported to date that combine: gene therapy + bacteria-based therapy + surgical removal of primary tumors, for the prevention and treatment of the metastatic disease, and that achieved long-term curative results in the clinically relevant 4T1 model. In addition, we demonstrated that all re-challenged mice were able to respond to a second neoadjuvant SFV-IL-12 + LVR01 treatment, rising further optimism about this experimental approach, since in the clinic, relapsed mammary tumors are often more difficult to be therapeutically solved.

This strategy would have at least three main theoretical advantages compared to other immuno- or chemical- based therapies. First, the *in situ* administration of each therapeutic agent would avoid, in theory, systemic toxicity. Second, an expected locally induced immunity would specifically recognize and destroy cancer cells, thus diminishing unspecific effects. Third, by combining SFV-IL-12 with *Salmonella* and surgery we could reduce the doses of the viral vector, more complex to produce than bacteria, lowering the cost of therapy. In addition, we believe that such a strategy could be clinically feasible because: (i) administration of alphavirus-based vectors similar to SFV, has been already proven to be safe in phase I/II clinical trials [65] (ii) live attenuated *Salmonella* strains have also been assayed in clinical trials showing a good safety profile [66] with a bacteria-based therapy successfully being used for the treatment of patients with bladder cancer [67, 68], (iii) neoadjuvant i.t. administration of a gene therapy vector combined with chemotherapy has been already clinically tested in LABC patients, showing to be feasible and safe [69]. Finally, combinatorial treatments are perceived as a major pathway for progress in cancer therapy with a number of pre-clinical and clinical events revealing synergistic antitumor activities. It is therefore encouraging to anticipate that combined biological approaches could become interesting options for the treatment of malignancies that lack cure in the present.

## Conclusions

We have correlated the metastatic 4T1 model with severe cases of locally advanced breast cancer in the clinic. In addition, we found that mice that underwent surgery of primary tumors 16 days after 4T1 cells inoculation could represent a good model for minimal residual disease, since at this time-point 100 % animals presented lung micro-metastasis which progressed until causing death in all animals. Aiming to find an effective treatment against metastatic breast cancer, we have developed a novel approach based on the combined intratumoral administration of a SFV vector expressing interleukin-12 (SFV-IL-12) and attenuated *Salmonella* LVR01 followed by surgical removal of the treated primary tumors. This neoadjuvant combined therapy was markedly synergistic compared to each treatment alone and its efficacy depended on the order in which both factors were administered. We achieved 90 % long-term survival only when SFV-IL-12 was administered previous to LVR01, whereas administration of LVR01 before SFV-IL-12 did not have any antitumor activity. Our observations point out to the importance of reducing tumor angiogenesis and inducing an efficient immune response at early stages of primary tumor establishment in order to prevent and/or eradicate disseminated 4T1 metastasis before surgical intervention. These antitumor effects were associated to early and sufficient IL-12 and INF-γ expression levels induced by SFV-IL-12 inoculation. We also found that, although the combined therapy does not induce an efficient long-term protection against 4T1 tumors, 100 % of re-challenged mice could be totally cured by applying the same neoadjuvant combined regimen. These data increase the clinical relevance of this experimental approach, since it could be applicable to patients who suffer from relapse. In addition, this study may provide new opportunities to evaluate such a biological therapy against a number of other aggressive and metastatic types of cancers.

## Additional files

Additional file 1: ***Salmonella*** **LVR01 persists and replicates into inoculated tumors.** (PDF 26 kb)
Additional file 2: **Relative amount of CD8+ and CD4+ T cells in lymph nodes isolated from animals treated with the effective and the ineffective combined therapy.** (PDF 92 kb)

## Abbreviations

MBC: Metastatic breast cancer
MRD: Minimal residual disease
LABC: Locally advanced breast cancer
IL-12: Interleukin-12
LacZ: b-galactosidase
SFV: Semliki Forest virus
vp: viral particles
cfu: colony forming units
PBS: Phosphate buffer saline
FBS: Fetal bovine serum
ELISA: Enzyme-linked immunosorbent assay
DMEM: Dulbecco’s modified eagle medium
PFA: Paraformaldehyde
DAPI: 4’,6-diamidino-2-phenylindole
i.t: intratumoral
IFN-γ: Interferon gamma
UdelaR: Universidad de la República
CIMA: Center for Applied Medical Research
