# Supplementary material for: Neoadjuvant administration of Semliki Forest virus expressing interleukin-12 combined with attenuated Salmonella eradicates breast cancer metastasis and achieves long-term survival in immunocompetent mice
Source: BMC Cancer. 2015 Sep 7;15:620. doi: 10.1186/s12885-015-1618-x (PMC4562361; doi:10.1186/s12885-015-1618-x)
Supplement: Additional file 1: — Salmonella LVR01 persists and replicates into inoculated tumors. (PDF 26 kb) [file 12885_2015_1618_MOESM1_ESM.pdf]

Salmonella LVR01 persists and replicates into inoculated tumors

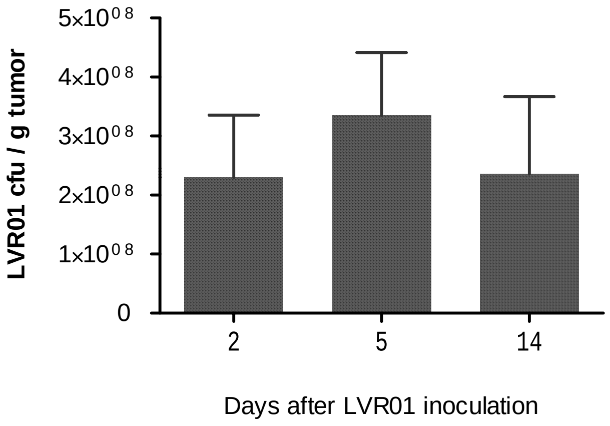

Description of data: $2x10^7$ bacterial units were resuspended in 50 ml PBS and administrated i.t in mice 10 days after orthotopical implantatation of 4T1 cells. Mice were sacrificed 2, 5 or 14 days later and tumors were collected, weighted and mechanical disaggregated. Serial dilutions of these tissue lysates were plated incorporated into LB agar. Plates were incubated at 37°C overnight to allow colony formation. Bacteria per gram were calculated taken into account the number of colony forming unit (cfu), the dilution factor and the weight of each tumor. Our data show that the number of inoculated bacteria increments with time and accompanies tumor growth.
