# Supplementary material for: Neoadjuvant administration of Semliki Forest virus expressing interleukin-12 combined with attenuated Salmonella eradicates breast cancer metastasis and achieves long-term survival in immunocompetent mice
Source: BMC Cancer. 2015 Sep 7;15:620. doi: 10.1186/s12885-015-1618-x (PMC4562361; doi:10.1186/s12885-015-1618-x)
Supplement: Additional file 2: — Relative amount of CD8+ and CD4+ T cells in lymph nodes isolated from animals treated with the effective and the ineffective combined therapy. (PDF 92 kb) [file 12885_2015_1618_MOESM2_ESM.pdf]

Relative amount of CD8$^+$ and CD4$^+$ T cells in lymph nodes isolated from animals treated with the effective and the ineffective combined therapy

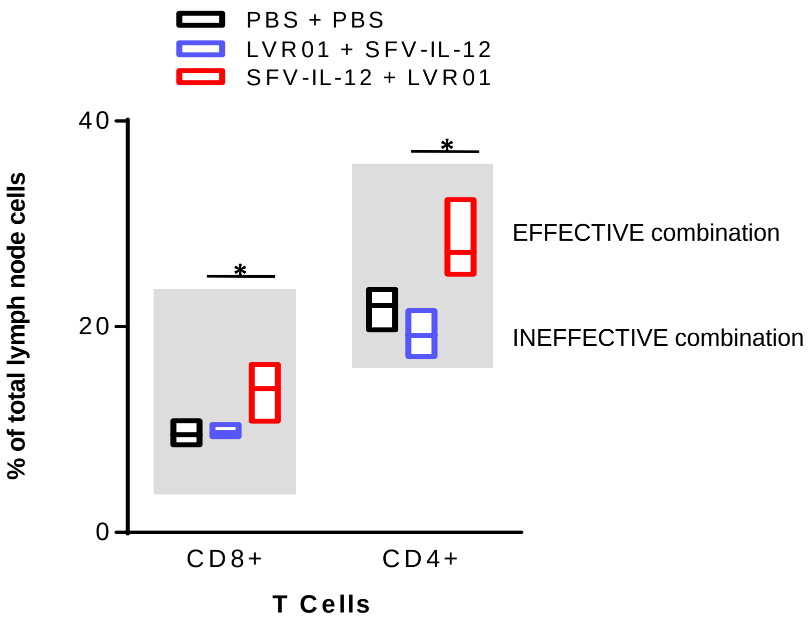

Description of data: Draining lymph nodes were obtained from treated and and control mice 3 days after the second i.t dose of PBS, vector (SFV-IL12) or bacteria (LVR01), respectively. Lymph nodes cells were mechanically disaggregated, washed with PBS, filtered through a 70 mm Falcon cell strainer and 10^6 cells were incubated with the correspondent conjugated antibodies mixture in conic bottom 96 well plates. CD8$^+$ T cells were identified by combined CD45-PE, CD3-PerCPCy5 and CD8-PerCp.Cy7 staining and CD4$^+$ T cells were identified by combined CD45-PE, CD3-PerCPCy5 and CD4-APC.Cy7 staining (BD Biosciences). Stained samples were fixed on 2% PFA, washed and read using FACS CANTO II cell cytometer. Doublets and cellular debris were excluded from analysis by means of gating, and 100,000 events were acquired per sample. Simple stains and unstained controls were used to adjust laser intensity and compensation between channels. Data were analyzed using FACSDIVA software (BD Biosciences) and show a significant higher amount of CD8$^+$ and CD4$^+$ T cells in draining lymph nodes isolated from SFV-IL-12 + LVR01 compared to LVR01 + SFV-IL-12 treated animals. $p < 0.05$ (*).
